# Opportunities to link Heart Failure Guidelines and chronic disease management—preliminary considerations

**DOI:** 10.31083/j.rcm2304142

**Published:** 2022-04-12

**Authors:** Pupalan Iyngkaran, Rosemary Calder, Craig Nelson, Judy Lowthian, Charlotte Hespe, John Horowitz, Maximilian P. de Courten

**Affiliations:** ^1^Mitchell Institute and Institute for Health and Sport, Victoria University, 3000 Melbourne, Australia; ^2^Medical School Werribee Campus, University of Notre Dame, 3030 Werribee, Australia; ^3^Division of Health Policy, Mitchell Institute, Victoria University, 3004 Melbourne, Australia; ^4^Division of Chronic and Complex Care, Western Health Chronic Disease Alliance, 3012 St Albans, Australia; ^5^Department of Nephrology, University of Melbourne, 3010 Melbourne, Australia; ^6^Department of Research, Bolton Clarke, 3131 Forest Hill, Australia; ^7^Department General Practice and Primary Care Research, Sydney School of Medicine, University of Notre Dame, 2010 Darlinghurst, Australia; ^8^Department of Cardiology, University of Adelaide, 5005 Adelaide, Australia

**Keywords:** chronic disease management, clinical guidelines, chronic heart failure, epidemiology, processes of care, systems of care

## Abstract

**Background:**

Enhancing community based Chronic Disease Management (CDM) will make significant impacts on all major chronic disease management outcome measures. There are no successful models of community hubs to triage and manage chronic diseases that significantly reduce readmissions, cost and improve chronic disease knowledge. Chronic heart failure (CHF) management foundations are built on guideline derived medical therapies (GDMT). These consensuses evidenced building blocks have to be interwoven into systems and processes of care which create access, collaboration and coordinate effective and innovative health services.

**Methods:**

Perspective and short communication.

**Conclusions:**

This review explores: (i) conventional chronic disease management in Australia; (ii) Possible options for future chronic diseases models of care that deliver key components of CHF management.

## 1. Introduction

Chronic diseases remain a challenge for all aspects of today’s Australian medical practice. Nearing two decades now, since the introduction of government initiatives such as *Keeping people well *(focus on Prevention) and *The Better Health Initiative* (*ABHI*), the Federal and States funded more than 500 million on five elements of chronic care including self-management, and more recently the National Strategic Framework for Chronic Conditions [1, 2, 3]. Chronic heart failure (CHF) is a chronic cardiovascular disease, along with other illnesses with chronic disease label makes up 85% of presentations to primary care [4], was incorporated in this. It remains unclear whether diseases should be classified under a chronic disease umbrella or under a disease umbrella and have a chronic disease angle. There have been tremendous gains and there remain some gaps.

In the next decades we will find that these gaps are a health services issue and could benefit from weaving individual diseases and its chronological phenotypes better. The burden in clinical and economic terms paint a picture that supports more innovation is required to address this issue. The lack of threads between clinical views and available models suggests that to some degree there are silos between the protagonists of this problem. As an example, the strength of guidelines and gaps in community outcomes highlights translational issues between evidence and uptake [5, 6, 7, 8, 9, 10, 11, 12]. This short piece is aimed at looking back at an Australian health systems perspective on CHF guidelines within a chronic disease model from the three points above: its burden, the models of care and clinical translational; traditional chronic disease management models that target readmission and cost effectiveness; and to explore possibilities for feasible, evidence-based methods to identify and target personalised lifelong care for prevention and management.

## 2. Epidemiology

The observation advancing HF as a subspeciality started authoritatively from early community studies such as Framingham study. Since then, improved diagnostics, an aging population the term *epidemic* was introduced as cases were increasingly diagnosed. It was becoming evident that this syndrome aligns a high burden on patients and health services, even before accounting contributing aetiologies such as ischemic heart disease and comorbid conditions such as diabetes, renal failure, in more than half of cases. This burden lies in the disease complexity, health resource utilisation especially a high readmission, life-long treatments, and regular multidisciplinary follow-up. The burden on health system includes epidemiology (see below); pathophysiology including race, renal function, coronary heart disease, rheumatic heart diseases, and alcohol; and *Health Care Gaps *or differential distribution of health resources [4] on the case mix across the health continuum. More than half of CHF patients suffering at least one other chronic condition, and between 4.5 to 11 million (10–20%) of Australian population have a one or more chronic condition [13].

The Australian landscape matches *global CHF epidemiology* [14, 15, 16, 17, 18, 19, 20, 21]. In Europe and globally, prevalence is >15 million and 37.7 million, respectively. In Asia the prevalence range is 1.3–6.7%, South America 1% and no data for Sub-Saharan Africa [17, 18]. In the United States, prevalence is >5.8 million, projected to be 8 million by 2030, with 870,000 new cases yearly, CHF being the lead cause for hospitalisation for those over 65 years of age, with >1 million primary presentations or 1% to 2% yearly. Annual Medicare expenditure in the US for HF is expected to rise from $20.9 (2012) to $53.1 billion (2030) [14]. Readmission is 20–30% in one month, 50% in 6 months and 80% of emergency presentations are admitted [15, 16, 17, 18]. Pay for performance or fee-for-service, health system or client focused models have not been universally effective [19, 20]. Diastolic Heart Failure or Heart Failure with preserved ejection fraction is 50% of CHF and >60% female. There appears little difference in phenotypes as most evidence suggest cost and readmission risk to be similar [14].

*HF data from Australia* [21, 22, 23, 24, 25, 26, 27, 28, 29] are derived from several sources, with no single comprehensive prospective dataset. Let us explore this from three categories:

(i) Burden of HF - Prevalence and incidence is based on AIHW estimates and applying overseas findings no studies reported on the incidence, applying overseas findings at 30,000 diagnosed new cases yearly [21]. The AIHW self-reported data above 18 years of age (two-thirds >65 years), estimates 0.5% (range from 1.0–2.0%) or >104,900 previously undiagnosed cases [4, 13, 23]. Chan *et al*. [21] highlights 480,000 [6.3% (95% CI 2.6–10.0) (66% men)] living with CHF. Morbidity and mortality for adults >65 years is high [29]. The pooled 30-day and 1-year all-cause mortality were 8% and 25% respectively [16]; readmission - 6-month readmission and five-year mortality at 50% [18]. There are variations with jurisdiction [21, 22, 23, 24, 25, 26]. Hospitalisations can cost between $900 to $2.7 billion from community to hospital care [21]. Limited data in rural patients and Indigenous people [28, 30, 31].

(ii) HF Programs – must navigate complex disease, high resource use and high burden on comorbid conditions. All these influence CHF outcomes. Screening and prevention, monitoring and treatment are integral [29].

(iii) Unmet needs – future projections point at significant increase in incidence, burden, and cost annually to >51,000 individuals; and prevalence of >1.5 million cases by 2030 and estimated annual cost of $3.8 billion [22]. Specific considerations have to be given to priority and vulnerable populations including elderly and Indigenous populations. These groups suffer disproportionately from disease burden, hospitalisations, non-traditional risk factors, and delays and uptake of guideline derived medical therapies (GDMT) [4, 31].

## 3. Models of care

Approaches that enhance community based chronic disease management (CDM) incorporate health system monitoring, consumer feedback and policy change to increase participation and improve health outcomes, will make significant impacts on all major CDM outcome measures. There are no successful models of community hubs to triage and manage chronic diseases that significantly reduce readmissions, cost and improve chronic disease knowledge [1, 2]; as building blocks do not constitute a system of care, access, care coordination and collaboration, effectiveness and innovation of new ideas are imperatives and must be enhanced [32, 33].

**Fig. 1.**
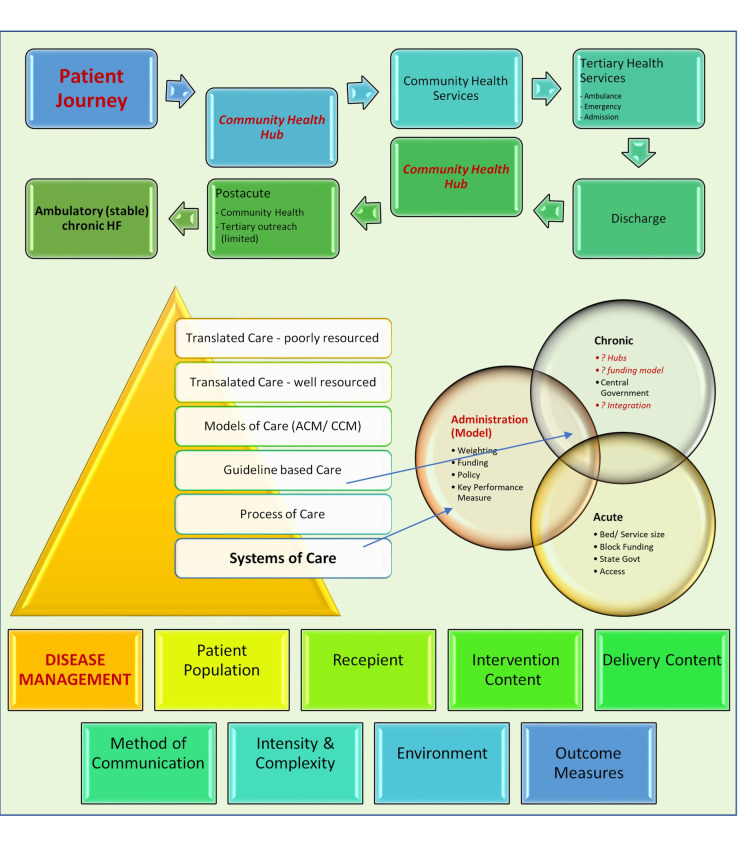
**Building blocks to navigate patient journeys**. Foundations of health systems of care are formulated from a system of care (pyramid). The building blocks of these include disease management systems. interaction of health administrators, formulated knowledge and health services. Health institutions play a critical role in patient journeys via a community health services or acute pathways. A missing component of this pathways a robust, integrated community health hubs that act as a buffer for some cases transitioning between acute and chronic phases. Abbreviations: ACM, acute care model; CCM, chronic care model [7].

The sentinel evidence and impetus for chronic care model (CCM) more than a quarter of a century highlighted that patient care requiring multiple services, show the greatest improvement in health outcomes on 4 pillars (Tables 1,2, Ref. [2, 7, 11, 34]): improving health services expertise and skill, patient support and education, team-based care planning and delivery, and enhanced health information systems and registries [34, 35, 36]. It is fundamental from the outset we define what is the key concept of a chronic illness. Chronic disease is also synonymous with complex disease; however, the two terms have different contexts for Australian Health system [12, 13]. Chronic disease rightly can be matched with disease specific management; however, complexity has less often been looked at as a logistics issues of which tools like chronic disease self-management (CDSM) could be beneficial. The American Heart Failure guidelines 2021 has seen introduction on several new pharmaceuticals with revolutionary benefits in HF. Simultaneously and unfortunately CDSM programs have been demoted from lack of evidence [4]. The CCM is well described [34, 35, 36], when models are looked at in chronological terms, the Acute Care Model(ACM) in can achieve comparable outcome rates as summarise in consensus guidelines (Fig. 1, Ref. [7]). In many well-resourced institutions, acute HF needs are met. It could be argued that in some instances gains in acute can be diminished by failure in some aspects of the community chronic HF care. This then adds an unpredictable readmission burden that diminishes the otherwise sound system of care.

**Table 1.** **Health systems terminology**.

Term	Definition
Alignment	Adoption of an existing system or process of care.
Burden	Disease or health problem impacts on communities measured by adverse events (mortality, morbidity), financial cost or other indicators. Can be quantified by of quality-adjusted life years (QALYs) or disability-adjusted life years (DALYs) or health budgets.
Collaboration	Developing a strategy to align systems or process of care that may or may not be similar.
Chronic care model	Clinical framework modelled on Wagner’s findings to design health service care delivery or disease management for chronic diseases. Six fundamental areas (extended model not detailed): (1) Health System – Organization of Healthcare; (2) Self-Management Support; (3) Decision Support; (4) Delivery System; (5) Design Clinical Information Systems; (6) Community Resources and Policies.
GDMT	Guideline Derived Medical Care (GDMT) are systematically (including consensus) developed statements to assist health practitioners on evidenced treatments and the level of evidence to provide appropriate care for defined clinical diagnosis e.g., congestive heart failure.
Health pathways	Care pathways are developed to manage patient care, improve quality, reduce variation, and increase efficient use of healthcare. They provide a mechanism for integrating evidence-based medicine into clinical practice.
Health systems	*Capacity Building*:
	∙ The development of knowledge, skills, commitment, structures, systems and leadership to enable effective health promotion.
	*Strengthening*:
	∙ Six core components or “building blocks”: (i) service delivery, (ii) health workforce, (iii) health information systems, (iv) access to essential medicines, (v) financing, and (vi) leadership/governance.
	*Function*:
	∙ Essential (4): provision of health care services, resource generation, financing, and stewardship.
	∙ Other: quality, efficiency, acceptability, and equity.
	∙ Domain – area of control or sphere of knowledge for one independent health system problem.
	∙ Patient journey (6) Community (Baseline), Prehospital (ambulance, primary care, nurse-on-call, specialist), Hospital Rapid access (emergency), Hospital Admission (ward, intensive care), Hospital discharge, Community (early post discharge).
	∙ Dimension – care variables that when integrated make up the health service domain of care, e.g., blood biochemistry or echocardiography for heart failure diagnostic domain.
Process of care	Process of care refers to an evidence-based action or intervention performed during the delivery of patient care. Process of care measures reflect a healthcare facilities’ ability to execute and comply with recommended best patient care practices.
System of care	Principle-guided approach to developing and sustaining systemic changes, e.g., spectrum of effective, community-based services and supports for defined population that is organized into a coordinated network, integrates service planning and coordination and management across multiple levels, culturally and linguistically competent builds meaningful partnerships with families and communities; and addresses their cultural and broader needs at service delivery, management, and policy levels, and has supportive management and policy infrastructure.

**Table 2.** **The health basket for acute and chronic heart failure**.

Care model		Disease management domains							
Service Chronology	Domains	Patient population	Recipient	Intervention content	Delivery Content	Method of communication	Intensity & complexity	Environment	Outcome measures
1.ACUTE	Tertiary/Quaternary	∙ All comers	∙ Accommodates Multiple	∙ Multiple	∙ Multiple	∙ Not relevant	∙ Modifiable	∙ Not relevant	∙ Good
2. CHRONIC	Health System or Organisation of Health ${\mathrm{Care}}^{@}$	∙ Gradient of availbility and quality	∙ Demographic variables	∙ Demographic variables	∙ Demographic variables	∙ Options variable	∙ Resource Variables	∙ Access and Geographical Variable	∙ Gradient
	∙ Clinical Information ${\mathrm{Systems}}^{\&}$								
	∙ Decision ${\mathrm{Support}}^{\#}$								
	∙ Delivery System ${\mathrm{Design}}^{\$}$								
	Community Policy	∙ Patient access can be defined in some jurisdictions	∙ Social-cultural determinants	∙ Less complex in some jurisdictions	∙ Probably face to face or conventional delivery methods	∙ As above	∙ Cost-effectiveness unclear - may be better resourced in some jurisdictions	∙ As above	∙ Influence on outcome more likely influenced by above
	∙ Self-management Support*								
	∙ Patient Resources^								
	Other Models	NA	NA	NA	NA	NA	NA	NA	NA
	∙ ICIC								
	∙ Stanford								
	∙ TCM								
	∙ CCM+ICCC								

The acute care model (ACM) role is designed to neutralise early threats to live. The resourcing for this is >80% of heath budgets for only 15% of total medical presentations. In ACM, management domains can be actioned with high performance under one umbrella (hospital). There are small overlaps when commencing chronic disease care in hospital. Taking this working model into the community, e.g., the *Chronic Care Model* requires many domains or possibly excellence in one critical domain to achieve the same performance in ACM. To execute broadly, disease management elements, requires productive interactions between, community policies and resourcing and health organisational structure. The ability of ACM to achieve this regularly against CCM is part of the jigsaw on this complex health services canvas (some concepts from [2, 7, 11, 34]). ***Abbreviations:*** CCM + ICCC, chronic care model & Innovative Care for the Chronic Conditions; ICIC, Improving Chronic Illness Care; NA, not available; TCM, Transitional Care mode. ***Definitions:*** @ Program planning that includes measurable goals for better care of chronic illness; # Integration of evidence-based guidelines into daily clinical practice; $ Focus on teamwork and an expanded scope of practice for team members to support chronic care; & Developing information systems based on patient populations to provide relevant client data; * Emphasis on the importance of the central role that patients have in managing their own care; ^ Developing partnerships with community organizations that support and meet patients’ needs.

Clinical guidelines are consensus opinion of evidence. Their value is a foundation to base decisions for care. Interpretation of evidence for individualisation is a complex issue and continues to evolve. Guidelines however are a guide but not a substitute for clinical decision taking. Guidelines are a guide to how to populate care dimensions for process of care but not they are care directives. We are in a unique position where few will question what are in the guidelines or the principles of chronic disease models of care. However, we are still exploring what permutations can come from these standards. Chronic disease care and CHF will evolve more likely within what is available and tailoring it toward the needs of each jurisdiction.

## 4. Building on what works

**Fig. 2.**
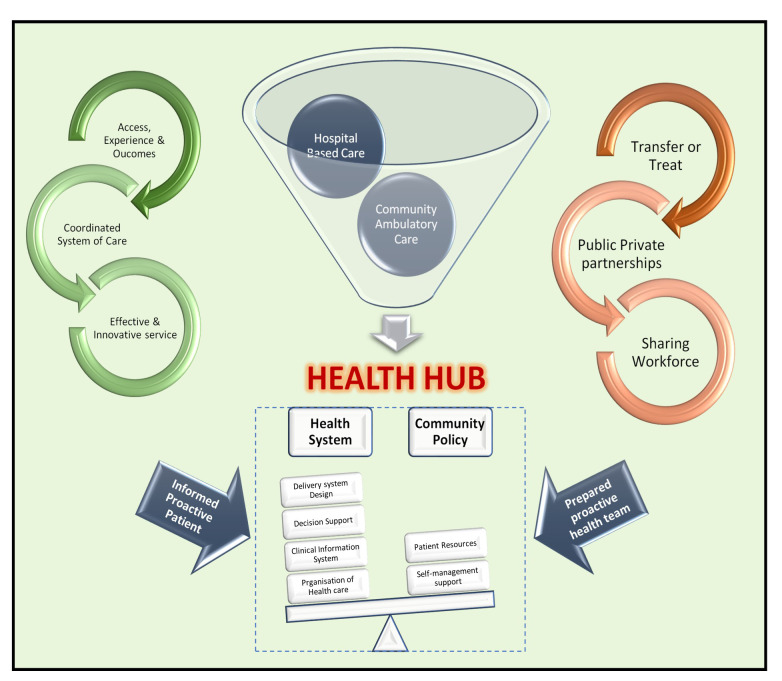
**Template for community chronic disease & rapid access hub**. Design, service and infrastructure plan white paper identifying key implementation areas for innovative cardiac services. Three core implementation points are identified on the left (green). Three core service points on the right (red), where health hubs must: firstly, navigate existing health system strengths for treating patients, however innovation could see greater range of care within communities; secondly, reform may be required to address questions of partnerships between private and public sectors and sharing of workforce across the health spectrum, which have been relatively untested [34, 42].

Understanding the epidemiology of HF, the established CCM’s are critical in delivering GDMT. Prescribing evidence classified as 1a, that provide the greatest proven avenues for improving NYHA class, morbidity and mortality, has post trial data and is undisputed [4]. Hospital process of care allows attainment of these outcomes across a wider population, is also undisputed [4, 7, 37, 38, 39]. There are gaps in chronic HF care, with gradients of care in being able to achieve most GDMT’s, and this is also undisputed [40, 41]. Importantly, as a collective however CHF has not seen a decline in morbidity and mortality similar to other cardiovascular diseases such as coronary artery disease, hypertension and rheumatic heart diseases [41]. We believe that identifying and investing in several community-based strategies could help shape a positive long-term solution. Several points are worth considering: firstly, does The Collaborative Model Improve Care for CHF [37]; secondly, does a life-long, illness with three phase trajectory (stable, decompensation, palliation) require increased patient self-management capacity and can this be achieved; thirdly, is a community health hub (Fig. 2, Ref. [34, 42]) concept that supports primary care, an adequate surrogate for hospital based programs of care [38, 40, 41, 43, 44, 45, 46, 47, 48, 49, 50, 51].

(i) The collaborative model of care works. There are numerous examples from chest pain services, acute care with components of ambulatory home-based care and heart transplant services. With all of these models there is usual a lead such as nurse led for home treatments or physician led for transplants. These examples however have a time limit. Community hub models with indefinite resourcing will require a mapping of resources and logistics to link collaborative opportunities.

(ii) Acquiring a lifelong illness has ripples in many directions. Navigating ones physical and mental self-care and bridging that to health services resources has great variations with any patient. Health systems have acknowledged the need to provide support, but it has never been clear how services are individualised to individual client needs. Furthermore, there are no risk scores that predict, and channel resources based on key factors such as vulnerability, readmission and mortality risk. More work is required to find a satisfactory solution here.

(iii) The community health hub that replicates on a smaller scale the acute infrastructure at an outpatient level and address the key cost-effectiveness measures of HF program, e.g., medications, self-management competence, rehabilitation and refreshers, checklist, and buffers non acute repeat clinical needs, consistently, at high quality for all comers in theory delivers a process of care similar to OPTIMIZE-HF and could be successful. In addition, these measures will support improved adherence, an understated but vital factor for improving outcomes [42].

If we are to build on what works in health systems is to understand fundamentally two factors: firstly, acute care has established success however requires concentration on resources; secondly, the chronic care model is theoretically sound, concentrate on patients that are ambulatory and require care across the health jurisdiction. The model that could fit this are *community-based health hubs with a rapid access service. *There are no funding models in Australian Healthcare for this. We thus believe these principals detailed in cardiac design white paper could help shape future direction [43]:

(i) Better patient access, experience and outcomes. Identification and management of HF risk is best in primary care. The management of acute care is often centralised and is quite advanced. The transfer to and from acute centres will respond to ongoing research and continuous quality improvement. Thus, primary care as the setting for most non-acute care, is appropriate with rising prevalence, aging multimorbidity populations [44], however adapting what works should revolve around the highly successful ACM. To provide patients with GDMT requires many of 8 domains and >35 care dimensions in disease management taxonomy. Thus, requires specialised units to be the focus rather than specialists to reside in multidisciplinary general practices.

(ii) Coordinated cardiac system of care – Hierarchy within services and across health subspecialities continues to be a challenging area in community practice. In reality the range of services outside acute care is spread over varied distances. In principle, with current technologies, virtual health hubs are conceivable, but they are exceptions with little published data in support. Carla *et al*. [47] investigated the primary care experience of >1000 older patients with chronic illness despite primary health professionals being important less than half had scheduled care plan and reminders. One in four had a good understanding of their medications.

(iii) Effective and innovative cardiac services – is the domain of research. Importantly phase-IV (post trial or translational) research is poorly done at a grass roots level. The ability to acquire evidence for a defined jurisdiction and implement it via policy is also not mainstay. No doubt identifying the jurisdiction that requires the investment in quality assurance, as opposed to accepting studies from global publication pool, so that findings would impact cost-effectiveness equations requires a strategy. We identify some areas below:

• Health economic institutes – highly skilled independent bodies to investigate health jurisdictions with lag in key performance measures and work with clinical teams on that area to explore relevant quality assurance questions.

• Policy and Funding – identify avenues for combined state and federal initiatives.

• Weighting – identifying methods to define jurisdiction case mix and loads.

• Performance outcome measures – reductions on readmissions and acute service workload that are preventable and through cost-effectiveness.

• Model – identify option that factor existing services and either align, integrating, collaborate or innovate service models.

• Turning around negative areas and resourcing keyroot factors – among 2082 participants CDSM was highest in developed than developing nations [45]. There are now vast demographics in developed nations and some of the areas requiring improved CDSM are those with LSES. These are also the areas that suffer inequitable provision of optimal HF services. Innovations are needed to bridge these outcome, resource, and socio-cultural barriers.

## 5. Conclusions

CHF as a chronic disease has had translational difficulties in chronic care. There are heterogeneity of factors that could be considered. To build on this we acknowledge strengths in systems of care, guidelines, models of care. In translation the acute care utilising a standardised disease management taxonomy has consistently provided strong performance on key measures. The system starts to falter when the focus moves in chronology (chronic care), and process of care adapts to care delivery outside the hospital umbrella. Interestingly all six domains of the CCM provide a foundation for this. The closest link to the acute model in achieving GDMT, that uses similar disease management domains, administered using CCM principles, appear the chronic care health hub. These services exist but are few and the processes of care are yet to be adequately described and standardised. With health budgets strained, there may not be goodwill for innovation and adding cost, thus of finding a fit among existing resources could be successful. We advocate a research focus in this area.

## Abbreviations

ACM, acute care model; AHF, acute heart failure; AMA, Australian Medical Association; CCM, chronic care model; CDM, chronic disease management; CDSM, chronic disease self-management; CHF, congestive/chronic heart failure; ED, emergency Department; GBMT, guideline based medical therapy; GP, general practitioner/practice; HF, heart failure; RCT, randomised controlled trial.
